# One new genus and three new species in the leafhopper tribe Coelidiini from the Neotropical Region (Hemiptera, Cicadellidae, Coelidiinae)

**DOI:** 10.3897/zookeys.794.26474

**Published:** 2018-11-01

**Authors:** Xiu-Dan Wang, Ya-Lin Zhang

**Affiliations:** 1 Key Laboratory of Plant Protection Resources and Pest Management of the Ministry of Education, Entomological Museum, Northwest A&F University, Yangling, Shaanxi, 712100, P. R. China Northwest A&F University Shaanxi China

**Keywords:** Auchenorrhyncha, Brazil, Homoptera, morphology, Peru, taxonomy

## Abstract

One new leafhopper genus *Paracodilia***gen. n.** with one new species *P.geniculata***sp. n.**, and two other new species in different genera, *Bolivielaexpanda* and *Armaturolidiasymmetrica***spp. n.**, are described in the tribe Coelidiini (Cicadellidae: Coelidiinae) from the Neotropical region. Photographs and illustrations are provided.

## Introduction

**Figures 1–9.**
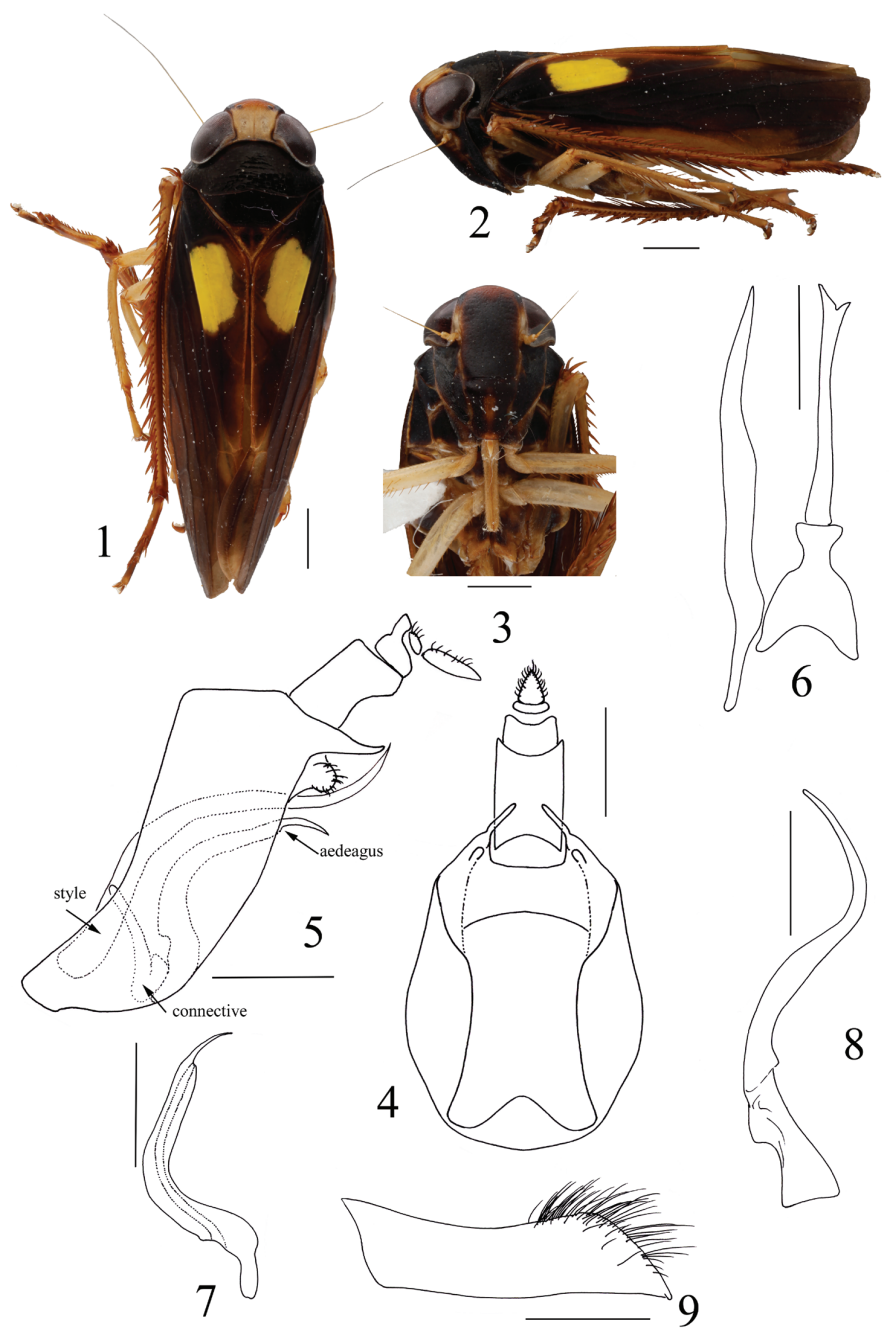
*Paracodiliageniculata* sp. n. Holotype: **1** habitus, dorsal view **2** habitus, lateral view **3** face **4** pygofer and segment X, ventral view **5** pygofer, aedeagus, connective, style and segment X, lateral view **6** aedeagus, connective and style, ventral view **7** aedeagus, lateral view **8** style, lateral view **9** subgenital plate, ventral view. Scale bars: 1 mm (**1–3**), 0.5 mm (**4–9**).

**Figures 10–20.**
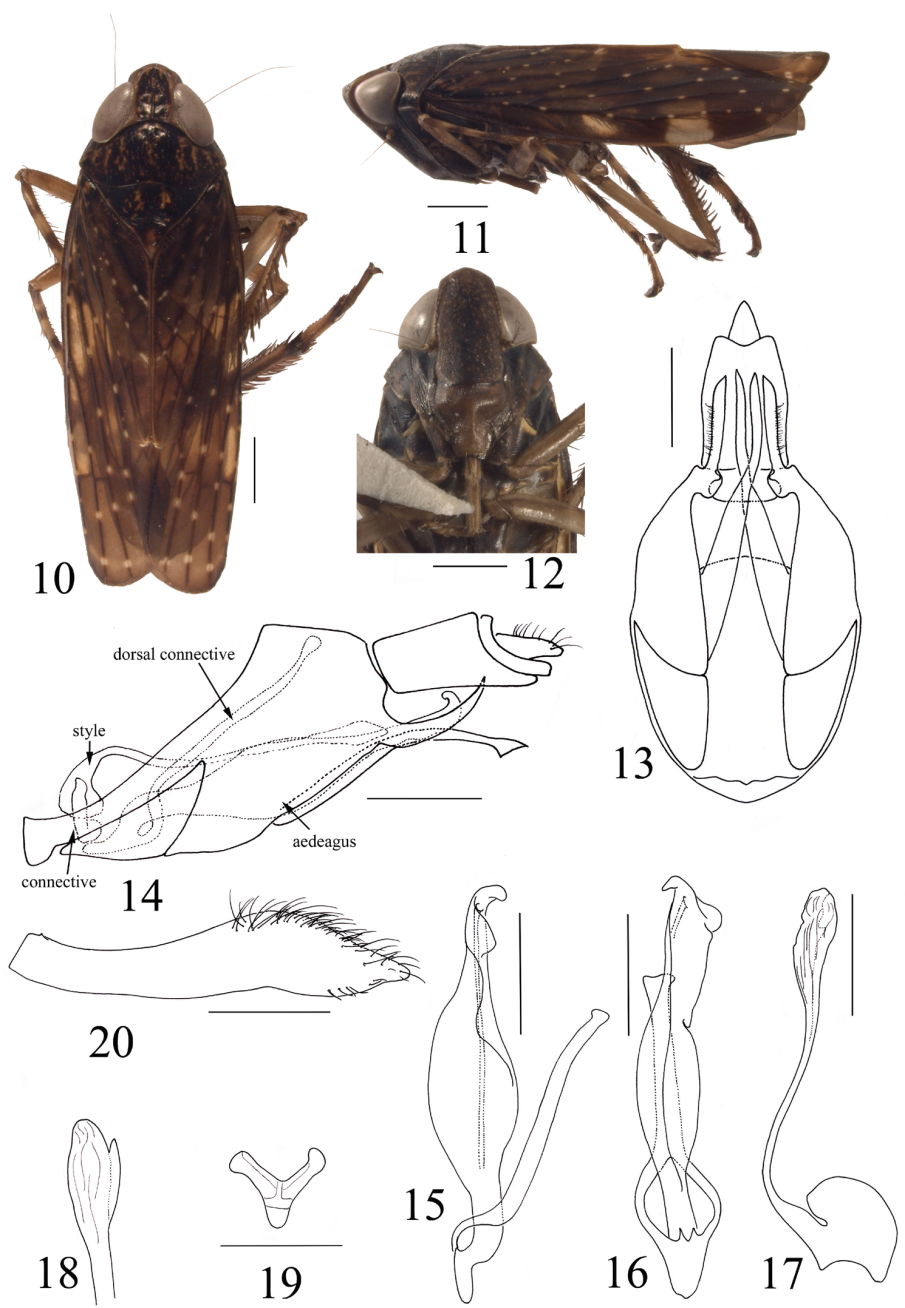
*Bolivielaexpanda* sp. n. Holotype: **10** habitus, dorsal view **11** habitus, lateral view **12** face **13** pygofer and segment X, ventral view **14** pygofer, aedeagus, connective, style, dorsal connective and segment X, lateral view **15** aedeagus and dorsal connective, lateral view **16** aedeagus and dorsal connective, dorsal view **17** style, lateral view **18** apex of style, ventral view **19** connective, caudal view **20** subgenital plate, ventral view. Scale bars: 1 mm (**10–12**), 0.5 mm (**13–20**).

**Figures 21–31.**
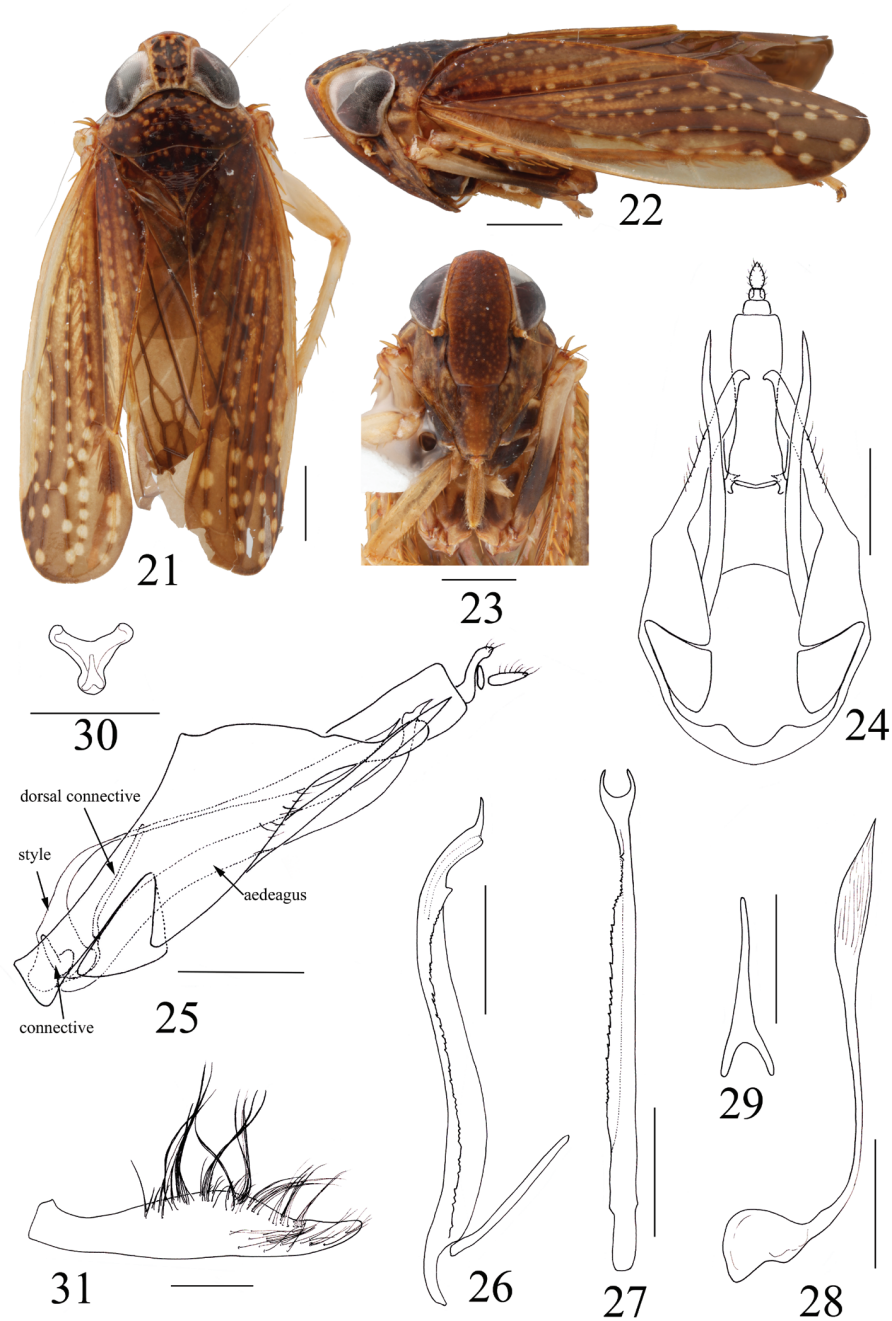
*Armaturolidiasymmetrica* sp. n. Holotype: **21** habitus, dorsal view **22** habitus, lateral view **23** face **24** pygofer and segment X, ventral view **25** pygofer, aedeagus, connective, style, dorsal connective and segment X, lateral view **26** aedeagus and dorsal connective, lateral view **27** aedeagus, dorsal view **28** style, lateral view **29** dorsal connective, dorsal view **30** connective, caudal view **31** subgenital plate, ventral view. Scale bars: 1 mm (**21–23**), 0.5 mm (**24–31**).

Coelidiinae is a large leafhopper subfamily found throughout the tropics. Its members are distinguished externally mainly by the very narrow vertex compared to the extremely large eyes (Figs 1, 10, 21) and long face with antennae situated near the lower corners of the eyes in facial view (Figs 3, 12, 23). In the Neotropics, there are six tribes of Coelidiinae, the largest being the Coelidiini, which includes half of the 70 known genera of the tribe (see Nielson 2011 for a key to the genera). In this paper, we describe and illustrate one new genus *Paracodilia* gen. n. with a new species, two new species of the genera *Boliviela* and *Armaturolidia*, and provide a brief discussion on the tribe from the region.

### Materials and methods

The male genitalia of the specimens examined were macerated in 10% NaOH and observed in glycerin jelly using an Olympus SZX10 stereomicroscope. All drawings were made using an Olympus drawing tube. A Canon EOS 5D Mark II camera and lifting table controlled by CAMLIFT V2.7.0 were using to take photographs at different focal planes and then stacked by ZERENE STACKER. Photos were also taken by a ZEISS SteREO Discovery.V20 stereomicroscope equipped with a ZEISS Axiocam ICc 5 camera that also provided measurements.

Terminology follows Nielson (1982), Nielson (2011), and Dietrich (2005). Measurements follow Wang and Zhang (2017). Type specimens are deposited in the Universidad de San Marcos, Lima, Peru (**USML**) and Universidad Federal do Rio de Janeiro, Brazil (**UFRJ**).

## Taxonomy

### Subfamily Coelidiinae Dohrn, 1859

**Diagnosis.** In addition to the external characters noted in the Introduction the following characters are diagnostic. Prothracic tibia with accessory row of setae between AD and AV setae rows. Forewing with three anteapical cells, usually with only the outer one closed, and appendix well developed and extended around the apex (Felix et al. 2002: 163, figs 6–9). Male genitalia with pygofer fused to valve and caudal corner produced slightly to strongly, often with processes or pair of lobes; connective Y-shaped; styles sometimes asymmetrical; dorsal connective usually present, attached dorsally to base of aedeagus, variable in length and shape (stick-like, Y-shaped, n-shaped or slightly bifurcate at apex). Female genitalia with valvulae long and slender, second valvulae with a prominent middorsal preapical tooth and additional asymmetrical teeth distally (Fan et al. 2015: 475, figs 81, 82; Viraktamath and Meshram 2017: 276, figs 29–31).

#### Tribe Coelidiini Dohrn, 1859

**Diagnosis.**Coelidiini can be differentiated from other tribes by the following combination of characters. Crown moderately long, midlength produced distally beyond anterior margin of eyes, distal length 1/4 to 1/3 of entire median length. Eyes large, occupying approx. 2/3 of entire dorsal area of head (more than 2/3 in Tinobregminiand less than 2/3 in Youngolidiini). Clypeus elongate, maximum width more than 1/2 but less than 3/4 of length, without median longitudinal carina or present but incomplete in a few species (complete in Teruliini). Pygofer triangle with 0-3 pairs of caudal processes. Dorsal connective usually present, stick-like, sometimes bifurcated at base or apex. Subgenital plate compressed, not segmented (depressed and segmented in Thagriini and Tharrini). Segment X slightly shorter to longer than pygofer dorsal length (very short in Thagriini).

##### Genus Paracodilia gen. n.

Taxon classificationAnimaliaHemipteraCicadellidae

http://zoobank.org/B1492322-E1A5-4E7D-A072-34256DDECF51

Figures 1–9

###### Type species.

*Paracodiliageniculata* sp. n.

###### Etymology.

The generic name refers to the similarity between the new genus and *Codilia* Nielson, 1982.

###### Diagnosis.

General color deep brown. Crown light brown with distal 1/4 orange, pronotum and mesonotum pitchy without spots; eyes piceous; forewing with distinct yellow mark on clavus, veins without spots (Figs 1, 2). Face fuscous (Figure 3).

Anterior margin of head obtusely angled. Crown midlength produced distally beyond eyes, approx. 1⁄3 of entire length; area between eyes as wide as eye width, surface rugulose, elevated above eyes; lateral margins slightly carinate, not surpassing anterior margin of eyes, slightly convergent basally. Medial length of pronotum distinctly longer than crown and quite shorter than mesonotum (Figure 1). Forewings typical of subfamily (Figure 2). Clypeus long and narrow, lateral margins slightly convex; clypellus broad, maximum width approx. equal to midlength, base inflated, lateral margins constricted medially (Figure 3).

###### Male genitalia.

Valve triangulate in ventral view. Pygofer narrow in lateral aspect, caudodorsal margin produced and tapered in lateral view, without triangular plates at base (Figure 4), caudoventral processes short, covered with short spines, adjacent to base of caudodorsal processes (Figure 5). Segment X elongate, lateral margins parallel in ventral view (Figure 4). Aedeagus moderately long and narrow, somewhat tubular, bent at midlength in lateral aspect, apex asymmetrically bifurcate; gonopore apical on ventral surface between bifurcations (Figs 6, 7); dorsal connective absent. Connective large, Y-shaped with stem broad, shorter than length of arms (Figure 6). Style very long, much longer than aedeagus, sinuate in lateral view, tapered to acute apex (Figs 6, 8). Subgenital plate narrow, lateral margin abruptly tapered distally with dense setae (Figure 9).

###### Remarks.

This new genus is similar to genus *Codilia* Nielson, 1982 externally, but has very different male genitalia. The aedeagus is distinctly curved at midlength in lateral view and apex asymmetrically bifurcate, but in *Codilia* the aedeagus is slightly curved with a large retrorse spine at the apex. Also in the new genus the subgenital plate is highly setose laterally in its apical half but sparsely setose in *Codilia*.

##### Paracodilia geniculata sp. n.

Taxon classificationAnimaliaHemipteraCicadellidae

http://zoobank.org/D79A3FE3-1B83-4F2A-8F8D-4007859EE270

Figures 1–9

###### Type material.

Holotype male, PERU: Pasco, 20 km N Villa Rica, 1600 m, 10°43'6"S 75°10'55"W, 21 October 2002, C. Dietrich (3371) [USML].

###### Diagnosis.

Pygofer with caudoventral processes short, covered with short spines, adjacent to base of caudodorsal processes; aedeagus somewhat tubular, bent at midlength in lateral aspect, apex asymmetrically bifurcate, gonopore apical on ventral surface between bifurcations, dorsal connective absent; style much longer than aedeagus, tapered to acute apex; subgenital plate with dense setae.

###### Description.

***Measurements.*** Male. Body length 7.59 mm; crown length 0.65 mm; width between eyes 0.84 mm; eye width 0.68 mm; clypeus length 1.52 mm; clypellus length 0.83 mm; pronotum length 0.74 mm; mesonotum + scutellum length 1.00 mm. Female unknown. External morphology and male genitalia as described in generic diagnosis.

###### Etymology.

The species epithet is derived from the Latin word *geniculus*, referring to the aedeagus being geniculate or bent medially in lateral view.

##### Boliviela expanda sp. n.

Taxon classificationAnimaliaHemipteraCicadellidae

http://zoobank.org/45AF317B-43E4-4866-9C09-7FFDF0804FD8

Figures 10–20

###### Type material.

Holotype male, PERU: Ancash Region, 4 km NW Carhuaz, Auquipampa, 9°16.91'S 77°39.23'W, 3640 m, 17–18 September 2009, coll. ME Irwin (1212) [USML].

###### Diagnosis.

Pygofer with caudodorsal margin produced as a curved, tapered process, caudoventral processes subbasally, apex slightly enlarged in lateral aspect, extended beyond caudodorsal processes; aedeagus large and broad, lateral edge rolled ventrally, tapered to compressed apical 1/4 in lateral view, apex curved dorsally, gonopore subapical on lateral surface, dorsal connective bifurcate at base, enclosing aedeagus subbasally; style reaching to apex of aedeagus, needle-like with apical third enlarged, corrugated, a small process at subapex; subgenital plate with dense hair-like setae at apical half.

###### Description.

***Measurements.*** Male. Body length 8.01 mm; crown length 0.88 mm; width between eyes 0.71 mm; eye width 0.69 mm; clypeus length 1.57 mm; clypellus length 1.00 mm; pronotum length 0.94 mm; mesonotum + scutellum length 1.15 mm. Female unknown.

***External morphology.*** Medium species, body slender. General color dark brown. Crown, pronotum and mesonotum deep brown with trivial light-brown spots, some gathered in patches; eyes piceous; forewing brown with pellucid markings, veins dark brown with pale spots (Figs 10, 11). Face fuscous with dull yellow tiny spots (Figure 12).

Anterior margin of head obtusely angular. Crown midlength produced distally approx. 1⁄3 of entire length; area between eyes approx. as wide as eye width, surface without wrinkles, elevated above level of eyes; lateral margins parallel, slightly carinate, surpassing anterior margin of eyes distally. Medial length of pronotum longer than crown but shorter than mesonotum (Figure 10). Clypeus long, narrow, lateral margins convex basally; clypellus broad, maximum width approx. equal to midlength, base inflated, lateral margins constricted medially, tapered apically (Figure 12).

***Male genitalia.*** Pygofer short, caudodorsal margin produced as a curved, tapered process (Figure 14), basally with a triangular plate on each side, caudoventral processes subbasally, very long, apex slightly enlarged in lateral aspect, extended beyond caudodorsal processes; segment X broad and long with lateral margins parallel in ventral view (Figure 13). Aedeagus large and broad, lateral edge rolled ventrally, tapered to compressed apical 1/4 in lateral view, apex curved dorsally; gonopore subapical on lateral surface; dorsal connective bifurcate at base, enclosing aedeagus subbasally (Figs 15, 16). Connective very small, Y-shaped, anterior stem very short (Figure 19). Style long, reaching to apex of aedeagus, needle-like with apical third enlarged, corrugated, a small process at subapex (Figs 17, 18). Subgenital plate narrow, outer lateral margin convex at apical third, tapered to apex, dense hair-like setae at apical half (Figure 20).

###### Etymology.

The specific epithet is descriptive of the expanded aedeagus in dorsal and lateral views.

###### Remarks.

The new species keys to *Collasuyusana* Nielson in Nielson (2011), but its aedeagal shaft is atypical of this genus. We therefore provisionally assign the species to *Boliviela* based on the inflated aedeagal shaft, despite the dorsal connective being atypical of this genus. According to Prof. Nielson, retired faculty of Brigham Young University (pers. com.) the aedeagus holds historically a higher diagnostic value than the dorsal connective.

##### Armaturolidia symmetrica sp. n.

Taxon classificationAnimaliaHemipteraCicadellidae

http://zoobank.org/01C8019E-94C7-46DD-8335-C517D3E57F34

Figures 21–31

###### Type material.

Holotype male, BRAZIL: MG, Santuario do Caraça, Cascatona Trl. 20.071°S, 43.488°W, 1076 m, 16 March 2015, CH Dietrich, sweeping forest understory (2211) [UFRJ].

###### Diagnosis.

Pygofer with caudodorsal margin moderately produced, apex rounded, with two long and narrow processes subbasally, surpassing caudodorsal processes, apex acute; aedeagus somewhat tubular, shaft with serrated flange from subbase to subapex laterally on right, apex symmetrical, bifurcated, gonopore dorsal at apex between bifurcations; style approx. equal to length of aedeagus, very narrow with apical third enlarged, surface rugulose, apex lanceolate; Subgenital plate with very long hair-like setae densely.

###### Description.

***Measurements.*** Male. Body length 7.6 mm; crown length 0.75 mm; width between eyes 0.73mm; eye width 0.88 mm; clypeus length 1.84 mm; clypellus length 0.99 mm; pronotum length 0.75 mm; mesonotum + scutellum length 0.76 mm. Female unknown.

***External morphology.*** Medium sized, slender species. General color brown. Crown light brown with dark brown marks; pronotum and mesonotum brown with pale spots; eyes gray; forewing with pale spots along veins (Figure 21); face brown with dense light brown spots on clypeus and clypellus (Figure 23).

Anterior margin of head angular. Crown in middle produced distally approx. 1⁄3 of entire length; area between eyes apparently narrower than width of eyes, surface without wrinkles, elevated above level of eyes; lateral margin slightly carinate, surpassing anterior margin of eyes distally, slightly convergent basally. Median length of pronotum about equal to length of crown and mesonotum (Figure 21). Clypeus elongate, lateral margins convex; clypellus narrow, basal width less than 2/3 midlength, base flattened and narrow, apex slightly expanded (Figure 23).

***Male genitalia.*** Pygofer with caudodorsal margin moderately produced, apex rounded; triangular plates at base of pygofer, two long and narrow processes subbasally, surpassing caudodorsal processes, apex acute; segment X elongate, slightly constricted medially in ventral view (Figs 24, 25). Aedeagus elongate, somewhat tubular, apical half recurved dorsally, shaft with serrated flange from subbase to subapex laterally on right, apex symmetrical, bifurcated; gonopore dorsal at apex between bifurcations (Figs 26, 27); dorsal connective very short, narrow, slightly bifurcate at base (Figure 29). Connective very small, Y-shaped, stem shorter than length of arms (Figure 30). Style very long, approx. equal to length of aedeagus, very narrow with apical third enlarged, surface rugulose, apex lanceolate (Figure 28). Subgenital plate narrow, outer margin convex medially and tapered to apex, with very long hair-like setae densely (Figure 31).

###### Etymology.

The species epithet is named for the aedeagus apex being symmetrically bifurcate.

###### Remarks.

The new species keys to genus *Armaturolidia* Nielson in Nielson (2011), but the general color of brown with pale marks is different from the original diagnosis of that genus (unicolorous or with black band). This species is close to *A.denticulata* Nielson, 1986 in some male genitalia characters but can be separated by the lateral margin of crown extending beyond eyes, caudodorsal processes of pygofer not bilobed, style equal to aedeagus in length and apex enlarged, and aedeagus apex symmetrically bifurcate.

## Discussion

The Neotropical Coelidiini fauna is very rich in general, and highly variable morphologically, e.g., in some genera such as *Coelidia* Germar the crown is elevated above the level of the eyes and its anterior margin is obtusely angulate while in some other genera such as *Spinolidia* Nielson the crown is approximately even with the eyes, slightly depressed medially with anterior margin broadly rounded and more similar to the Afrotropical and Oriental species. Two of genera treated here (*Boliviela* and *Armaturolidia*) belong to the *Boliviela* genus group which also comprises *Collasuyusana* Nielson, *Ventrolidia* Nielson, *Carinoscapula* Nielson, *Paralidia* Nielson, *Dicodia* Nielson, *Gracilidia* Nielson and *Tinocripus* Nielson. This group is diagnosed by a narrower angulate head with elevated crown. Also, most species in the group have the male pygofer with separate triangular plates basally and long ventral processes subbasally (Figs 13, 24). The former, previously neglected, character is also found in some Youngolidiini such as *Youngolidia* (see Nielson 1992: figs 25, 34, 37) and *Rikana* (see Nielson 1992: fig 1), suggesting a close relationship between the two tribes. A similar plate is also present in the cicadellid subfamily Hylicinae, e.g., *Kalasha* Distant and *Hemisudra* Schimdt, but the relationship between these characters in these taxa is unexplored.

## Supplementary Material

XML Treatment for Paracodilia

XML Treatment for Paracodilia geniculata

XML Treatment for Boliviela expanda

XML Treatment for Armaturolidia symmetrica
